# Aroma Characterization of Gardenia Black Tea Based on Sensory Evaluation and Headspace Solid-Phase Microextraction–Gas Chromatography–Mass Spectrometry

**DOI:** 10.3390/foods14234022

**Published:** 2025-11-24

**Authors:** Shenghong Zheng, Hongling Chai, Chunju Peng, Qi Huang, Mingkun Xu, Xingjun Wen, Huajing Kang

**Affiliations:** 1 Wenzhou Key Laboratory of Early-Sprouting Tea Breeding, Wenzhou Academy of Agricultural Sciences, Wenzhou 325006, China; zsh1418@126.com (S.Z.); chaihl2021@163.com (H.C.); chunjupeng@163.com (C.P.); huangqi@wzvcst.edu.cn (Q.H.); 2 Hanchá (Cangnan) Agricultural Development Co., Ltd., Wenzhou 325006, China; 13868852529@163.com; 3 Lishui Wenji Tea Industry Co., Ltd., Lishui 323000, China; wxztea@163.com

**Keywords:** gardenia black tea, aroma characterization, HS-SPME-GC-MS, OPLS-DA analysis, rOAV value

## Abstract

Gardenia black tea (GBT) is a prized Chinese scented tea, renowned for its pleasant aroma. However, the influence of repeated scenting rounds on its volatile profile remains poorly characterized. This study investigated the aroma profiles of GBT produced with zero, two, and three scenting rounds (T0, T1, T2) using sensory evaluation and Headspace Solid-Phase Microextraction–Gas Chromatography–Mass Spectrometry (HS-SPME-GC-MS). Sensory analysis revealed that GBTs (T1, T2) scored significantly higher in aroma and taste than the black tea base (T0). GC-MS analysis identified numerous volatile compounds, with esters, terpenoids, and ketones being predominant. Multivariate analysis identified 52 key volatiles (VIP > 2.0) that differentiated the tea samples. Among these, 28 compounds had odor activity values (OAVs) > 1, indicating significant sensory contributions. Ultimately, 11 volatiles, including (*Z*)-hex-3-enyl acetate, linalool, and (*E*)-hex-2-enal, were identified as the crucial basis for the characteristic fresh and floral scent of GBTs. The specific abundance levels of these compounds are hypothesized to underlie the superior, fresh, and elegant aroma of T1, compared to the slightly ripe and stuffy floral notes of T2. Therefore, it is advisable to prioritize two rounds of scenting during the production of gardenia black tea. These findings provide a theoretical foundation for optimizing the scenting process and enhancing the quality of GBT.

## 1. Introduction

Tea ranks among the most widely consumed non-alcoholic beverages globally. It is primarily categorized into six main types, namely green tea, black tea, oolong tea, yellow tea, white tea, and dark tea, each distinguished by its unique processing techniques [1]. Scented tea, a traditional reprocessed tea from China with a history spanning more than a millennium, is highly valued for its aromatic qualities [2]. This tea is created by infusing tea bases, such as green, black, or oolong tea, with a variety of flowers, resulting in well-known varieties including jasmine, magnolia, osmanthus, and gardenia tea [3]. Scented tea is primarily categorized by the type of flowers used, including jasmine, magnolia, osmanthus, and gardenia, among others. Currently, jasmine green tea is the dominant product in Chinese floral teas, both from a consumer and research perspective [4,5,6,7]. In recent years, gardenia flowers have gained popularity for their elegant and sweet fragrance, which imparts a refined and rich aroma to gardenia tea, characterized by a sweetness that is not overwhelming [8]. This has been a significant factor in the rise of new-style gardenia-infused beverages. However, to date, research on the aromatic compounds in gardenia tea is notably insufficient. A previous study identified 24 key odorants from gardenia green tea based on odor activity value ≥ 1 and GC-O-MS results [9]. However, the gardenia tea and samples were obtained from a market, and there is limited background information on the origin or processing methods. As a result, the research does not offer guidance for production or help improve gardenia tea quality. A separate investigation employed the combined Solid-Phase Microextraction and Gas Chromatography–Mass Spectrometry (SPME-GC-MS) technique to analyze the aroma components of GBT, identifying six relatively high-content components that may be the key contributors to the intense gardenia floral scent of tea [2]. Nevertheless, the study merely performed a basic comparison and lacked in-depth analysis. Similarly, a comparative study [10] employing HS-SPME-GC-MS, sensory evaluation, and ROAV analysis identified 10 key volatiles in Fuding Gardenia White Tea, which were established as quality assessment criteria for new gardenia white tea. To date, research on gardenia tea aroma has focused on the final product, leaving the important connection between processing methods and aroma quality largely unexplored.

Numerous factors can influence the quality of scented tea, such as the quality of the flowers, the tea base, the scenting technique (including methods and technological parameters), and so on [11,12,13]. In scented tea production, one of the most critical steps is blending flowers with tea leaves [14]. Typically, floral scenting is performed in two or three cycles to ensure aroma absorption. Compared with two rounds, three rounds allow more extensive interaction between the tea and flowers, theoretically facilitating greater uptake of aromatic compounds and thus a more pronounced floral fragrance in the final product [15]. However, additional scenting rounds also prolong production time, increase costs, and intensify labor requirements. Moreover, as aroma absorption involves both physical and chemical adsorption processes, repeated scenting not only enhances fragrance assimilation but also drives transformations in aromatic and flavor constituents, leading to discernible quality differences in gardenia-scented teas with different numbers of scenting rounds [16]. Tea quality is commonly assessed based on appearance, aroma, flavor, and liquor color, with aroma being a primary criterion—particularly for scented teas [17]. Therefore, it is essential to systematically evaluate the aroma profiles of GBT produced with varying numbers of scenting rounds.

Aroma quality is a critical determinant of the flavor and market value of scented tea [18]. Traditionally, the evaluation of aromatic properties in scented teas has relied heavily on sensory assessment, which is inherently subjective and lacks scientific data support [19]. Moreover, due to the complexity and instability of aroma compounds, sensory evaluations often yield inconsistent results. Therefore, it is essential to identify characteristic aroma substances to enable rapid and accurate discrimination of scented teas. The headspace SPME-GC-MS (HS-SPME-GC-MS) is a powerful technique for the qualitative and quantitative analysis of volatile components. It operates without damaging the sample and offers high sensitivity, rapid analysis, and solvent-free operation [20,21,22]. This method has been widely applied in tea research [23,24,25,26]. In recent years, chemometrics has become an essential strategy for extracting meaningful information from large datasets in the field of food flavor, including tea aroma. By integrating mathematics, statistics, and computer science, chemometrics maximizes the extraction of target information from complex data. Commonly used techniques such as principal component analysis (PCA), partial least squares-discriminant analysis (PLS-DA), orthogonal PLS-DA (OPLS-DA), and correlation analysis have been extensively employed in screening differential flavor compounds in tea [27,28]. This study employed HS-SPME-GC-MS coupled with multivariate statistical analysis to identify key volatile aroma compounds that distinguish scented GBT from Congou black tea. It further evaluated the changes in these aroma compounds across different scenting rounds. This study aims to characterize and compare the aromatic components across various scenting rounds of GBT, thereby providing a theoretical basis for optimizing the manufacturing process and enhancing GBT quality.

## 2. Materials and Methods

### 2.1. Materials and Reagents

The base for making the scented black tea was Congou black tea made from fresh leaves of the local tea tree variety harvested in late April 2024. The tea was scented with gardenia flowers collected during their full bloom in early June 2025, following the flower tea processing technique to produce gardenia-scented black tea. Following the traditional flower-tea scenting process, the gardenia scenting process is shown in Figure S1. The Congou black tea and gardenia flowers were sourced from Hanchá (Cangnan) Agricultural Development Co., Ltd. (Wenzhou, China), which is a company specializing in the production of herbal health teas.

The scenting process of gardenia flower tea was conducted in June 2025. Using raw black tea as the control (denoted as T0), two scenting-round treatments were set up: a double-round and a triple-round scenting process (denoted as T1 and T2, respectively). For each round of the scenting process, the number of flowers used was 30% of the raw black tea (the same ratio in the second and third rounds). Static scenting was conducted for approximately 15 h, then the samples were oven-dried at 105 °C until the moisture content reached about 8%, followed by the next scenting round. After scenting, the in-process tea was dried at 105 °C for 1.5 h. The moisture content of the GBT was controlled to be below 6%.

Sodium chloride (analytical grade) was purchased from Sinopharm Chemical Reagent Co., Ltd. (Shanghai, China). n-hexane (chromatographic grade) was purchased from Merck, Darmstadt, Germany. Standards were prepared using n-hexane and stored at −20 °C (chromatographic grade) from BioBioPha/Sigma-Aldrich, Saint Louis, MO, USA.

### 2.2. Sensory Evaluation

Each tea sample (3.0 g) was brewed with boiling water (150 mL) for 3 and 5 min, respectively, in a covered bowl at room temperature (RT, 25 ± 2 °C), according to the standard method for flower tea brewing, as described in Chinese standard GB/T 23776-2018 [29]. Thereby, tea samples were evaluated and scored through a traditional sensory evaluation process by 5 well-experienced tea sensory evaluation experts (holding achieved certificates for tea quality evaluation from the Tea Scientific Society of China) according to the China national standard on traditional sensory evaluation of tea (GB/T 23776-2018). The final score was obtained by averaging the scores from 5 panelists.

### 2.3. Instrument and Equipment

The n-hexane (internal standard) was obtained from Sigma-Aldrich (Shanghai, China). The names, types, and suppliers of analytical instruments and equipment are listed in Table S1.

### 2.4. Sample Preparation and Extraction by Headspace Solid-Phase Microextraction (HS-SPME)

Materials were harvested, weighed, immediately frozen in liquid nitrogen, and stored at −80 °C until needed. Samples were ground into a powder in liquid nitrogen.

Extraction procedure: 500 mg of tea powder was weighed into a 20 mL headspace vial, followed by the addition of a saturated sodium chloride (NaCl) solution, and then 20 μL of an internal standard solution (n-hexane at 10 μg/mL) was injected. The sample was then extracted using automated HS-SPME before proceeding to GC-MS analysis.

HS-SPME Parameters: The samples were stirred for 5 min at a steady temperature of 60 °C. Then, a 120 µm DVB/CAR/PDMS (Agilent Technologies Inc., Santa Clara, CA, USA) extraction head was placed into the headspace of the sample vial and extracted for 15 min. Before sampling, the extraction head was conditioned for 5 min at 250 °C in a Fiber Conditioning Station, followed by GC-MS analysis for separation and identification. It is important to note that new extraction heads are conditioned for 2 h in the Fiber Conditioning Station before use. The SPME Arrow used in this study provides a sensitivity ten times greater than traditional SPME fibers. All samples were tested in triplicate, with results expressed as mean ± standard deviation (SD).

### 2.5. GC-MS Conditions

After sampling, desorption of the VOCs from the SPME Arrow coating was performed in the injection port of the GC instrument (Model 8890; Agilent, Santa Clara, CA, USA) at 250 °C for 5 min. The identification and quantification of VOCs was conducted using an Agilent Model 8890 GC and a 7000 D mass spectrometer (Agilent, Santa Clara, CA, USA), equipped with a 30 m × 0.25 mm × 0.25 μm DB-5MS (5% phenyl-polymethylsiloxane) capillary column. Helium served as the carrier gas at a linear velocity of 1.2 mL/min. The injector temperature was maintained at 250 °C. The oven temperature was programmed starting from 40 °C (hold for 3.5 min), then increasing at 10 °C/min to 100 °C, at 7 °C/min to 180 °C, and at 25 °C/min to 280 °C, followed by a hold for 5 min. Mass spectra were recorded in electron impact (EI) ionization mode at 70 eV. The quadrupole mass detector, ion source, and transfer line temperatures were set at 150 °C, 230 °C, and 280 °C respectively. The MS was operated in selected ion monitoring (SIM) mode for the identification and quantification of analytes.

### 2.6. Qualitative and Quantitative Analysis of Volatile

The total ion chromatogram of the aroma components in tea samples was obtained under the optimized GC-MS conditions. Then, qualitative mass spectrometry analysis of the volatile compounds in the samples was conducted based on the MWGC database, a specialized database established with self-collected samples, which was derived from the NIST library and co-constructed with standard substances. Subsequently, the MassHunter quantification software (version 12.0) was employed to process the mass spectral files of the samples, selecting quantitative ions for the integration and calibration of chromatographic peaks. The quantification of substances was performed by calculating the ratio of the peak area of each detected volatile compound to that of the internal standard (n-hexane) for relative quantification. The estimation equation is as follows.

$$X_{i}=\frac{V_{s}\;\times \;C_{s}}{M}\;\times \;\frac{I_{i}}{I_{s}}\;\times \;10^{-3}$$

X_i_ represents the content of compound i in the sample (μg/g); V_s_ is the volume of the internal standard added (μL); C_s_ is the concentration of the internal standard (μg/mL); M is the mass of the sample (500 mg); I_s_ is the peak area of the internal standard; I_i_ is the peak area of compound i in the sample.

### 2.7. Odor Activity Values (OAVs) Calculation

The relative odor activity value (rOAV) is a method established by combining the sensory threshold of compounds to determine the key flavor compounds in foods. It is used to estimate each compound’s contribution to the overall aroma profile of the sample. In recent years, rOAV has been increasingly applied by scholars to identify the key flavor compounds in various foodstuffs. Generally, a rOAV ≥ 1 indicates that the compound directly contributes to the flavor of the sample. Based on the reported literature [30,31], the calculation formula for rOAV is as follows.

$$\mathrm{rOA}V_{i\;}=\;\frac{C_{i}}{T_{i}}$$

In the formula, rOAV_i_ represents the relative odor activity value of compound i, C_i_ denotes the relative concentration of the compound (in μg/g or μg/mL), and T_i_ stands for the threshold of the compound (in μg/g or μg/mL), which refers to the value measured in an aqueous matrix.

### 2.8. Data Analysis

The peak areas of all detected compounds were normalized to the total peak area of each sample to reduce systematic errors. Then, the normalized data were mean-centered and scaled to unit variance (UV scaling) before performing multivariate statistical analysis. Statistical analysis was conducted using one-way analysis of variance (ANOVA) with SPSS 22 software (IBM, Armonk, NY, USA). All comparisons were considered statistically significant if the *p*-value was less than 0.05. Orthogonal partial least squares discriminant analysis (OPLS-DA) was performed using SIMCA 14.1 (Umetrics, Umea, Sweden). Heatmaps and bar graphs were generated using Origin2025b software (Originlab, Northampton, MA, USA).

## 3. Results and Analysis

### 3.1. Sensory Evaluation of Black Tea Base and GBTs

**Figure 1:**
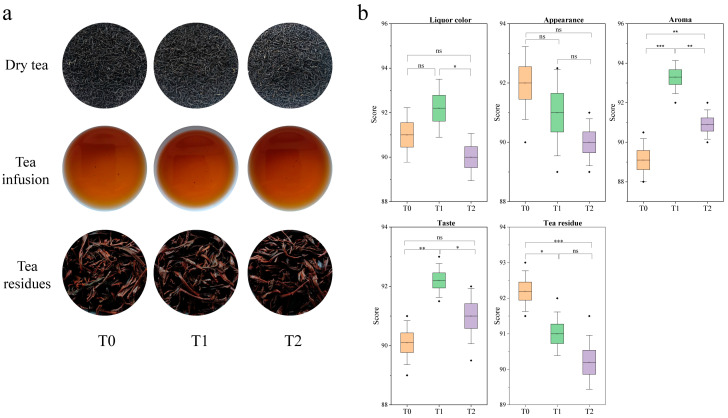
Results of sensory evaluation of black tea and GBTs. (**a**) Appearances of dry tea, tea liquor and infused tea. (**b**) Sensory scores. All values are mean ± SD, ‘*’, ‘**’ and ‘***’ indicate significant difference at *p* < 0.05, *p* < 0.01 and *p* < 0.001, respectively. And ‘ns’ means no significant difference between treatments.

The sensory evaluation results of tea samples indicated significant differences between raw black tea and GBTs. The appearance of raw black tea is compact, uniform, and glossy, with a deep reddish liquor and bright, even red infused leaves. The appearance of GBT with two rounds of scenting is relatively compact, uniform, and glossy, with a bright reddish liquor and bright, relatively even red infused leaves. The GBT with three rounds of scenting is characterized as relatively compact and somewhat uniform, with a somewhat bright reddish liquor and somewhat bright, somewhat even red infused leaves (Figure 1a). The scoring analysis results showed that the average score for the appearance of the raw black tea is 92 ± 1.22, higher than that of the GBTs (T1, T2) at 91 ± 1.46 and 90 ± 0.79, respectively (Figure 1b). In terms of liquor color, T1 scored the highest average of 92.2 ± 1.30, surpassing the T0 tea’s 91 ± 1.22 and significantly (*p* < 0.05) higher than the T2 tea’s 90 ± 1.06. T1 also achieved the highest average aroma score of 93.3 ± 0.84, significantly higher than T2 (90.9 ± 0.74) and extremely significantly higher than T0 (89.1 ± 1.08). Similarly, T1 scored the highest in flavor at 92.2 ± 0.57, significantly and extremely significantly (*p* < 0.01) higher than those of T2 (91 ± 0.93) and T0 (90.1 ± 0.74), respectively. For the infused leaves, T0 scored the highest at 92.2 ± 0.57, significantly and extremely significantly higher than those from T1 (91 ± 0.61) and T2 (90.2 ± 0.76), respectively (Figure 1b).

### 3.2. Characterization of Volatile Compounds in Black Tea Base and GBTs

#### 3.2.1. Identification of Volatile Compounds in Black Tea Base and GBTs

**Figure 2:**
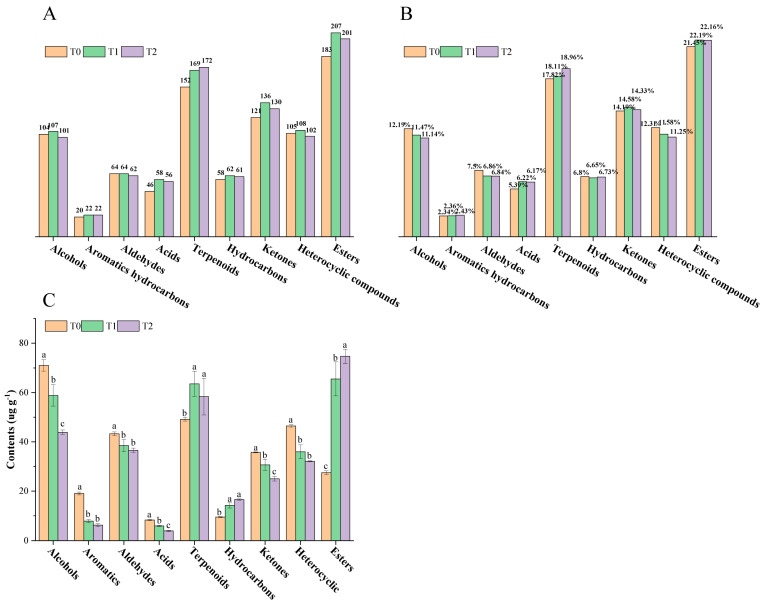
Information on volatile compounds in black tea base and GBTs. (**A**) Number of volatile categories both in black tea base and GBTs. (**B**) Proportion of volatile categories in black tea base and in GBTs. (**C**) Content comparisons of volatile categories in black tea base and GBTs. Different lowercase letters (a, b and c) indicate significant difference at *p* < 0.05.

Aroma is one of the most important determinants of overall tea quality [32]. Thus, a total of 920 volatile compounds (including 183 esters, 152 terpenoids, 121 ketones, 105 heterocyclic compounds, 104 alcohols, 64 aldehydes, 58 hydrocarbons, 46 acids and 20 aromatics hydrocarbons) were detected in black tea base (T0), while 968 volatile compounds (including 207 esters, 169 terpenoids, 136 ketones, 108 heterocyclic compounds, 107 alcohols, 64 aldehydes, 62 hydrocarbons, 58 acids and 22 aromatics hydrocarbons) were detected in T1, and 943 volatile compounds (including 201 esters, 172 terpenoids, 130 ketones, 102 heterocyclic compounds, 101 alcohols, 62 aldehydes, 61 hydrocarbons, 56 acids and 22 aromatics hydrocarbons) were detected in T2 by HS-SPME-GC-MS (Figure 2A). Esters, terpenoids and ketones were the dominant categories in triple samples, which accounted for 21.45%, 17.82% and 14.19% in T0, 22.19%, 18.11% and 14.58% in T1, 22.16%, 18.96% and 14.33% in T2, respectively (Figure 2B). More information about the total ion chromatograms of volatile compounds in T0, T1 and T2 is included in Figure S2.

Alcohols were the main volatile compounds, showing the highest content in T0 (71.01 ± 2.28 μg/g), while esters were the main volatile compounds, showing the highest content in T1 and T2, reaching 65.54 ± 6.86 μg/g and 74.70 ± 2.81 μg/g, respectively (Figure 2C). Terpenoids were the second largest category both in raw black tea (T0) and in GBTs (T1 and T2) (Figure 2B,C). The contents of terpenoids in T1 (63.54 ± 5.09 μg/g) and T2 (58.38 ± 7.46 μg/g) were equally significantly higher than that in T0 (49.15 ± 0.72 μg/g) (*p* < 0.05). Besides esters and terpenoids, hydrocarbons also showed significantly higher content in GBTs (T1 and T2) than that in raw black tea (T0), while alcohols, aromatic hydrocarbons, aldehydes, acids, ketones and heterocyclic compounds exhibited in the other way round, which were evenly present in significantly higher content in T0 than those in T1 and T2 (Figure 2C).

#### 3.2.2. OPLS-DA Analysis in the Volatile Compounds

**Figure 3:**
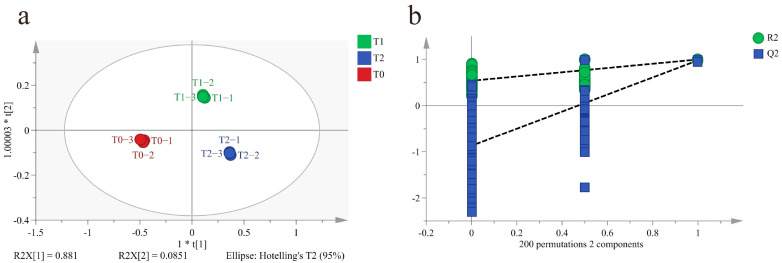
OPLS-DA results of black tea base (T0) and GBTs (T1 and T2) using GC-MS. (**a**) Score plot of OPLS-DA (R^2^Y = 0.999, Q^2^ = 0.989). (**b**) Plot of 200-permutation test (R^2^Y = 0.554, Q^2^ = −0.822).

To characterize the differences among T0, T1 and T2, an OPLS-DA model was constructed. A data matrix of 982 (volatile compounds) × 9 (samples) was obtained. A clear discrimination among T0, T1, and T2 was obtained (Figure 3a). The parameters of the model (R^2^Y = 0.999, Q^2^ = 0.989) demonstrated its good explanatory and predictive ability. To verify the robustness of the model, 200-permutation tests were carried out. The result showed that R^2^ and Q^2^ were (0, 0.554) and (0, −0.822) respectively, indicating that the model was robust and there was no overfitting (Figure 3b).

#### 3.2.3. Analysis of Differential Volatile Compounds in Black Tea Base and GBTs

**Figure 4:**
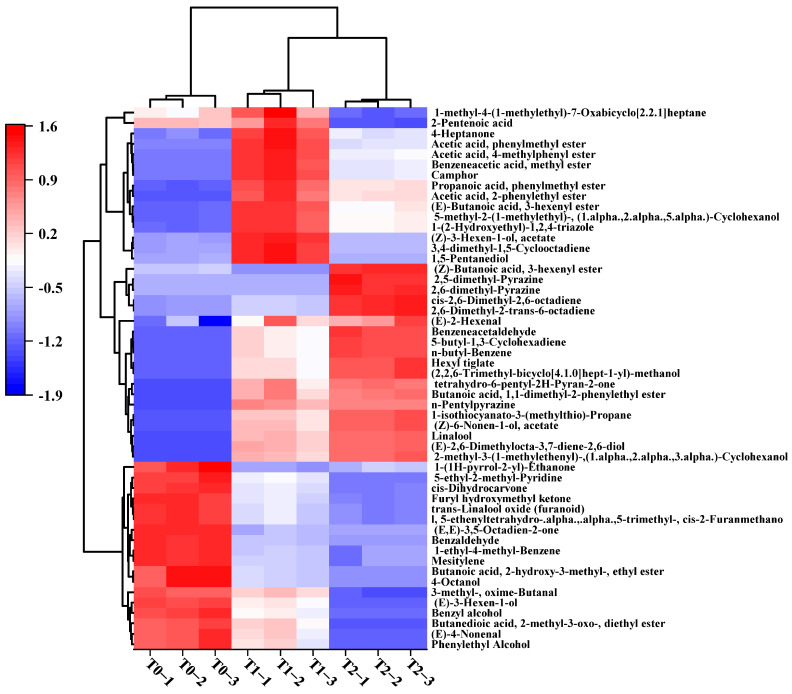
Heatmap constructed with 52 key differential volatiles with VIP > 2.0 in black tea base (T0) and GBTs (T1 and T2).

To further explore the key volatile compounds that differentiate raw black tea and GBTs, the variable importance in projection (VIP) was analyzed. Generally, a VIP value above 1 indicates that the variable plays a crucial role in group classification. In this study, 52 volatile compounds with VIP > 2.0 were identified. To visualize the differences among T0, T1, and T2, a heat map analysis was performed, and the results are shown in Figure 4. Clearly, the red color in the squares indicates higher content, while blue indicates lower content. Eighteen volatile compounds, such as 2-phenylethanol, phenylmethanol, octan-4-ol, (*E*)-hex-3-en-1-ol, 1,3,5-trimethylbenzene, 1-ethyl-4-methylbenzene, (*E*)-hex-2-enal, Benzaldehyde, (*E*)-non-4-enal, 3-methyl-oxime-Butanal, cis-Dihydrocarvone, and 1-(furan-2-yl)-2-hydroxyethanone, showed higher levels in T0 compared to T1 and T2. Conversely, some typical compounds like Linalool, (*Z*)-non-6-en-1-yl acetate, Camphor, 5-ethyl-2-methylpyridine, 2-Pentylpyrazine, (*E*)-Hex-2-enal, 2-phenylacetaldehyde, and Butylbenzene exhibited higher contents in GBTs (T1, T2) than in raw black tea (T0). Compared to T2, T1 had higher levels of 15 compounds, including Heptan-4-one, 4-methylphenyl acetate, (*Z*)-hex-3-enyl acetate, and 1,5-Pentanediol, while T2 showed elevated levels in 19 compounds such as Linalool, (*E*)-hex-2-enal, (*Z*)-Non-6-enyl acetate, (3*E*,6*E*)-octa-3,6-dien-2-one, Propane, 2-phenylacetaldehyde, 2-Pentylpyrazine, and Butylbenzene.

#### 3.2.4. OAV Analysis of Volatile Compounds in Black Tea Base and GBTs

**Figure 5:**
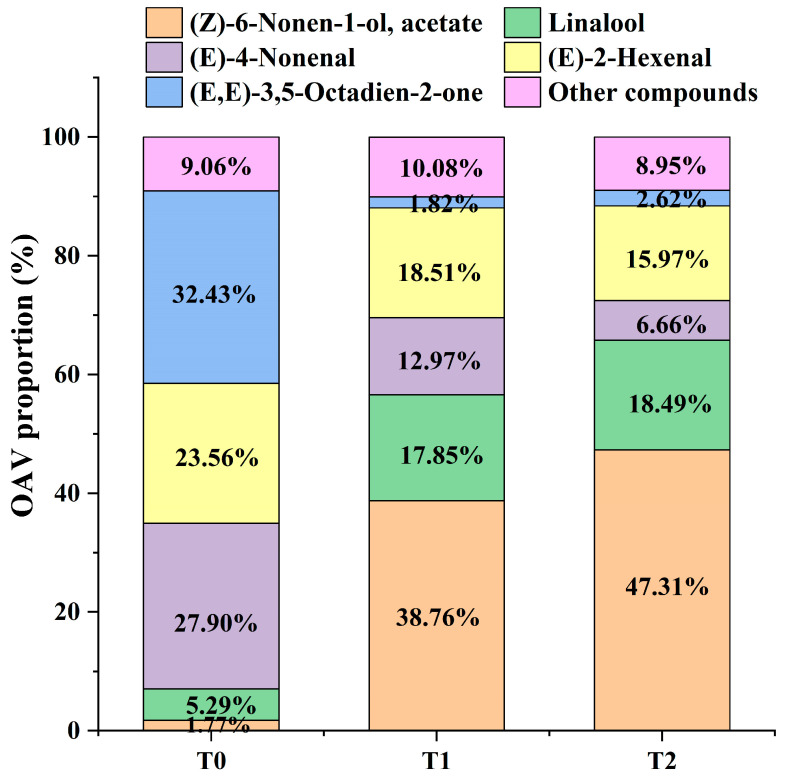
OAV proportion of the main volatile compounds in T0, T1 and T2.

OAV was an important indicator for assessing the contribution of volatile compounds to the overall aroma. Generally, volatile compounds with OVA ≥ 1 are considered key contributors responsible for the entire aroma profile. In this study, based on reported aroma component thresholds and attribute descriptions from the literature [33], the OVA values of 52 differential aroma components in raw black tea and GBTs were calculated. Using a cutoff of OVA greater than one, 28 differential aroma compounds were further selected (Table 1 and Table S2). Among these, 2-phenylethyl acetate (OVA = 0.64 ± 0.02), 6-pentyloxan-2-one (OVA = 0.47 ± 0.10), and Benzyl acetate (OVA = 0.31 ± 0.01) were the only three compounds with OVAs less than one in black tea base (T0), while the remaining 25 volatile compounds exhibited OVAs greater than one in both raw black tea and GBTs. Additionally, phenylmethanol (OVA = 116.83 ± 4.96) and 1-ethyl-4-methylbenzene (OVA = 207.66 ± 2.90) showed OVAs exceeding 100 in T0 compared to T1 and T2. Moreover, (3*E*,6*E*)-octa-3,6-dien-2-one (OVA = 4501.85 ± 17.52) displayed an OVA greater than 1000 in T0 versus T1 and T2. Notably, (*Z*)-6-Non-6-en-1-yl, acetate (OVA-T1 = 8994.41 ± 1004.62, OVA-T2 = 13,404.36 ± 567.38) and Linalool (OVA-T1 = 4129.04 ± 229.15, OVA-T2 = 5236.77 ± 83.02) had OVAs above 1000 in gardenia black tea compared to T0. Conversely, (*E*)-non-4-enal (OVA-T0 = 3871.69 ± 167.59, OVA-T1 = 3005.28 ± 239.69, OVA-T2 = 1886.61 ± 11.87) and *(E)*-hex-2-enal (OVA-T0 = 3275.61 ± 439.77, OVA-T1 = 4280.98 ± 360.41, OVA-T2 = 4524.17 ± 256.97) had OVAs exceeding 1000 in both raw black tea (T0) and GBTs (T1 and T2). Furthermore, the proportions of OAVs for (*Z*)-6-Nonen-1-ol and its acetate, as well as Linalool, increased significantly—from 1.77% and 5.29% in T0 to 38.76% and 17.85% in T1, respectively—and further to 47.31% and 18.49% in T2. Conversely, the levels of *(E)*-non-4-enal, (*E*)-hex-2-enal, and (3*E*,6*E*)-octa-3,6-dien-2-one decreased from 27.90%, 23.56%, and 32.43% in T0 to 12.97%, 18.51%, and 1.82% in T1, and further to 6.66%, 15.97%, and 2.62% in T2, respectively (Figure 5). Therefore, these compounds are considered to play a significant role in differentiating the aroma characteristics between GBTs and black tea base.

#### 3.2.5. Analysis of Key Aromatic Compounds in GBTs

**Figure 6:**
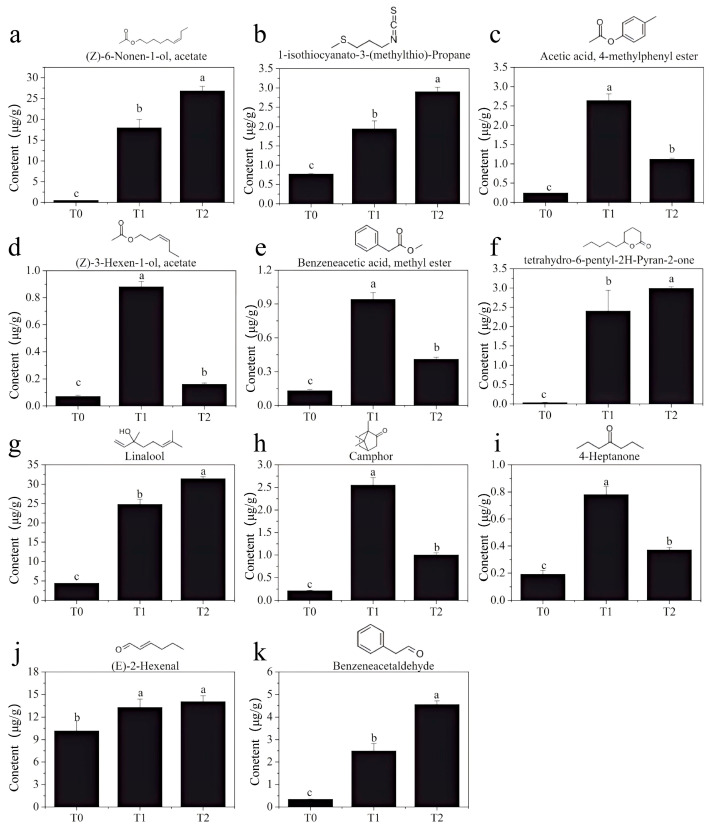
Structure and contents of 11 key volatile compounds (**a**–**k**) that are significantly higher in GBTs (T1 and T2) than in the black tea base (T0). Different lowercase letters (a, b and c) indicate significant difference at *p* < 0.05.

The key to manufacturing scented tea is ensuring that the raw tea fully absorbs the fragrance of fresh flowers, thereby improving the aroma quality. To further explore the characteristic aroma components of GBTs, a comparative analysis was conducted on 28 key aroma compounds, based on the significant increases observed in the GBTs after scenting. As a result, 11 aroma compounds were identified: (*Z*)-6-Non-6-en-1-yl, acetate, 1-isothiocyanato-3-methylsulfanyl propane, 4-methylphenyl acetate, (*Z*)-hex-3-enyl acetate, methyl 2-phenylacetate, 6-pentyloxan-2-one, linalool, camphor, heptan-4-one, (*E*)-hex-2-enal, and 2-phenylacetaldehyde. All these compounds have distinct floral and fruity aromas, forming the essential basis for the fresh and vibrant floral scent of gardenia tea. The chemical structural formulas and statistical analysis results are shown in Figure 6. It is clear that the contents of these 11 volatile compounds in GBTs (T1 and T2) are significantly higher than in raw black tea T0. Among them, linalool, (*Z*)-6-Non-6-en-1-yl acetate, and (*E*)-hex-2-enal have the highest concentrations in the GBTs, each exceeding 13 μg/g. 6-pentyloxan-2-one and (*Z*)-6-Non-6-en-1-yl acetate show the greatest increases in scented tea, with increases of more than 80-fold and 36-fold, respectively. Additionally, five compounds—4-methylphenyl acetate, (*Z*)-hex-3-enyl acetate, methyl 2-phenylacetate, Camphor, and heptan-4-one—have significantly higher contents in T1 than in T2. Conversely, six compounds—(*Z*)-6-Non-6-en-1-yl acetate, 1-isothiocyanato-3-methylsulfanylpropane, 6-pentyloxan-2-one, Linalool, (*E*)-hex-2-enal, and 2-phenylacetaldehyde—are significantly higher in T2 than in T1 (except for (*E*)-hex-2-enal). It is believed that these compounds may form the primary structural basis for the fresh and elegant aroma of the two-rounded scented GBT, which is superior to the slightly ripe and stuffy floral scent of the triple-time scented GBT.

## 4. Discussion

In this study, sensory evaluation showed that the overall appearance of GBT is slightly lower than that of raw black tea, but its aroma and flavor are significantly better than the latter (Figure 1). This is mainly due to the blending process of tea and flowers during scented tea production, which causes the tea body to break and reduces uniformity. In the subsequent scenting process, the tea leaves absorb the aroma of fresh flowers through moisture, which makes the leaves coarser, darker, and less glossy. However, the floral aroma absorption greatly improves the tea’s fragrance, characterized by fresh, elegant floral notes. This matches earlier research indicating that black tea may have a less appealing appearance, with duller, darker leaves, but excels in aroma and flavor. The improvements are due to the blending and scenting processes, which disrupt the tea matrix and help the tea absorb fresh floral volatiles [34,35].

In terms of volatile contents, it is clear that esters were the main volatile compounds in GBTs (Figure 2C). Among these, (*Z*)-6-Non-6-en-1-yl, acetate, Hexyl tiglate, and 4-methylphenyl acetate were the three most abundant volatile compounds with the highest content in T1 and T2. The results were similar to previous findings explored by Xie et al. [9]. Terpenoids were the second largest category after esters, showing significantly higher content in T1 and T2 compared to T0. Among them, linalool was the most abundant volatile compound in T1 and T2. It is widely known that linalool is associated with citrus-like and floral aromas [36,37]. Therefore, the scenting process is very important to the quality of scented tea. Alcohols had the third highest content in T1 and T2, significantly contributing to the sweet, floral, and grassy aromas found in tea [36]. Among these, 2-phenylethanol and phenylmethanol were the most and second most abundant volatile compounds, reported as important components in black tea and oolong tea, with rose-like and fruity aromas [30,38]. It is noteworthy that alcohol was the main volatile category in T0, with 2-phenylethanol and phenylmethanol showing significantly higher levels than in T1 and T2. This aligns with the results of previous studies [9]. The findings further support earlier research suggesting that, in the production of scented tea, the reduction in alcohol compounds and the increase in ester compounds are key factors influencing aroma changes [39,40,41].

Through multivariate statistical analysis (OPLS-DA, rOAV) combined with an essential scenting process that absorbs the fragrance of fresh flowers, the research identified 11 volatile compounds shown in Figure 6. These include (*Z*)-6-Non-6-en-1-yl acetate, 1-isothiocyanato-3-methylsulfanylpropane, 4-methylphenyl acetate, (*Z*)-hex-3-enyl acetate, methyl 2-phenylacetate, 6-pentyloxan-2-one, linalool, camphor, heptan-4-one, (*E*)-hex-2-enal, and 2-phenylacetaldehyde. All of these compounds are present in significantly higher amounts in T1 and T2 than in T0. They undergo notable changes during the processing of scented teas, resulting in a shift in the aroma profile of the tea base from original floral and tea-like notes to a more pronounced floral and fruity fragrance [42,43]. Collectively, they provide green-herb, fruity-sweet, floral, nutty-roasted, cool-herbaceous, and almond-like notes. Their combined presence enhances the tea’s fresh, bright, and complex aroma profile while contributing sweetness, fruitiness, and a characteristic scented-tea fragrance [44]. The findings also indicate that the overall quality of T1 exceeds that of T2 (Figure 1). Aromatically, T1 exhibits a fresh and elegant fragrance indicative of high quality, whereas T2, which shows a pronounced floral scent, tends to be somewhat dull and heavy, and its quality does not match that of T1. Based on the analysis of aromatic components, it is clear that the contents of (4-methylphenyl) acetate, (*Z*)-hex-3-enyl acetate, methyl 2-phenylacetate, camphor, and heptan-4-one are significantly higher in T1 than in T2, while the levels of linalool, 1-isothiocyanato-3-methylsulfanylpropane, (*Z*)-6-Non-6-en-1-yl, acetate, 6-pentyloxan-2-one, (*E*)-hex-2-enal, and 2-phenylacetaldehyde are notably lower in T1 compared to T2. Previous studies have shown that (4-methylphenyl) acetate and methyl 2-phenylacetate contribute sweet and ester-like characteristics [42,43], while (*Z*)-3-hexen-1-ol-acetate offers a fresh, green herbaceous fragrance [37]. Additionally, camphor imparts a cool, camphoraceous herbal aroma [43,44], and heptan-4-one provides a buttery or creamy sweetness [45,46]. Moreover, the green-herb and leafy notes from (*Z*)-6-Non-6-en-1-yl acetate and (*E*)-hex-2-enal lend freshness [42]. Linalool and 2-phenylacetaldehyde give dominant floral and sweet almond tones [9]. 1-isothiocyanato-3-methylsulfanylpropane introduces a mild spicy and sulfurous edge, and 6-pentyloxan-2-one adds a soft roasted and nutty background [45]. Collectively, these compounds shape the characteristic aroma of scented teas.

Compared to T1, T2 involved more scenting cycles. However, it is hypothesized that the black tea base may have reached a saturation point due to the complete absorption of floral aromas during the first two scenting cycles. This saturation likely caused a significantly reduced capacity of the tea leaves to absorb aromas during the third cycle, resulting in an aroma intensity in T2 similar to that of T1. This idea aligns with previous reports [47,48] and has been confirmed by pseudo-second-order kinetics and Langmuir/Freundlich isotherm models, which showed that aromatic adsorption reached equilibrium within a few hours, with the capacity leveling off, thus supporting the saturation hypothesis [49]. Additionally, the extended piling time during the third scenting round may promote anaerobic conversion of aromatic compounds, which can dull the aroma and reduce its freshness [16,50]. Therefore, with regard to production cost, labor, and product quality, two rounds of scenting are clearly better than three. Consequently, it is recommended to prioritize two rounds of scenting in the production of gardenia black tea. The findings of this study provide important theoretical insights and practical guidance for the scenting process of GBT.

## 5. Conclusions

This study characterized the volatile profiles of GBT using sensory evaluation and HS-SPME-GC-MS. Sensory analysis showed clear differences between GBT and the raw tea base, with the scented variants (T1 and T2) scoring significantly higher in both aroma and taste than the control (T0). We identified 920, 968, and 943 volatile compounds in T0, T1, and T2, respectively, with esters, terpenoids, and ketones as the main chemical classes in all samples. Multivariate analysis revealed 52 volatile compounds with VIP values > 2.0 as key to differentiating the samples. Of these, 28 compounds had OAVs above 1, indicating they are sensory relevant. Eleven of these were found to be essential to GBT’s fresh and vibrant floral aroma, with higher concentrations in T1 and T2 than in T0. Five of the eleven—including (4-methylphenyl) acetate, (*Z*)-hex-3-enyl acetate, methyl 2-phenylacetate, camphor, and heptan-4-one—were present at higher levels in T1 compared to T2. The remaining six—comprising (*Z*)-6-Non-6-en-1-yl, acetate, 1-isothiocyanato-3-methylsulfanylpropane, 6-pentyloxan-2-one, linalool, (*E*)-hex-2-enal, and 2-phenylacetaldehyde—were more abundant in T2. These compositional differences likely contribute to T1’s fresher, more elegant aroma, versus the riper, slightly stuffier floral character of T2. The findings provide a theoretical foundation and practical references for optimizing the process and improving the quality of gardenia tea.

## Figures and Tables

**Table 1:** OAVs (above 1) and VIP values (above 2) of differential aroma components among treatments.

No.	Compounds	CAS	Threshold (μg/g)	ROAV Value			VIP Value	Odor
				T0	T1	T2		
1	(*Z*)-6-Non-6-en-1-yl, acetate	76238-22-7	0.002	245.8 ± 11.8c	8994 ± 1004b	13404 ± 567a	7.68	Refreshing fruit, melon, honeydew
2	1-isothiocyanato-3-methylsulfanyl propane	505-79-3	0.005	15.36 ± 2.55c	388.2 ± 40.9b	580.6 ± 23.2a	2.52	earthy, vegetable, horseradish
3	4-methylphenylacetate	140-39-6	0.025	9.62 ± 0.13c	105.6 ± 6.5a	44.92 ± 1.38b	4.19	narcissus, phenol, animalic
4	(*Z*)-hex-3-enyl acetate	3681-71-8	0.031	2.44 ± 0.07c	28.31 ± 1.28a	5.05 ± 0.24b	2.80	fresh, green, sweet, fruity, banana, apple, grassy
5	methyl benzeneacetate	101-41-7	0.060	2.15 ± 0.13c	15.71 ± 1.06a	6.90 ± 0.27b	2.47	floral, honey, spice, waxy, sweet
6	2-phenylethyl acetate	103-45-7	0.249	0.64 ± 0.01c	3.75 ± 0.34a	2.46 ± 0.08b	2.05	rose, honey, tobacco
7	6-pentyloxan-2-one	705-86-2	0.066	0.47 ± 0.10b	36.32 ± 8.31a	45.33 ± 0.55a	2.65	creamy, coconut, fruity
8	Benzyl acetate	140-11-4	0.364	0.31 ± 0.01c	3.09 ± 0.25a	1.14 ± 0.05b	2.84	sweet, floral, fruity, jasmine, fresh
9	Linalool	78-70-6	0.006	733.8 ± 4.7c	4129 ± 229b	5236 ± 83a	7.92	floral, green
10	Camphor	76-22-2	0.016	13.24 ± 0.25c	159.3 ± 10.6a	62.30 ± 3.18b	4.22	camphor
11	(*E*)-non-4-enal	2277-16-9	0.002	3871 ± 167a	3005 ± 239b	1886 ± 12c	3.51	fruity
12	(*E*)-hex-2-enal	6728-26-3	0.003	3275 ± 439b	4280 ± 360a	4524 ± 257a	2.88	green, grassy
13	5-ethyl-2-methylpyridine	104-90-5	0.019	472.2 ± 18.9a	233.9 ± 14.4b	102.6 ± 0.7c	3.98	nutty, strong, raw, potato, roasted, earthy
14	2-[(2R,5R)-5-ethenyl-5-methyloxolan-2-yl]propan-2-ol	34995-77-2	0.190	18.09 ± 0.35a	10.98 ± 0.60b	8.32 ± 0.25c	2.06	flowery
15	2-pentylpyrazine	6303-75-9	0.005	45.43 ± 1.97b	478.7 ± 49.9a	511.1 ± 5.2a	2.60	-
16	(3*E*,6*E*)-octa-3,6-dien-2-one	30086-02-3	0.001	4501 ± 17a	421.1 ± 32.9c	740.1 ± 84.4b	2.45	fruity, green, grassy
17	heptan-4-one	123-19-3	0.008	22.33 ± 3.68b	98.81 ± 6.43a	92.67 ± 4.34a	2.17	fruity, cheese, sweet, cognac, pineapple
18	1-(furan-2-yl)-2-hydroxyethanone	17678-19-2	1.000	3.14 ± 0.06a	1.89 ± 0.07b	1.79 ± 0.08b	2.05	-
19	2-phenylacetaldehyde	122-78-1	0.006	54.18 ± 1.17c	395.4 ± 52.8b	722.6 ± 26.3a	3.25	floral, honey, rose, cherry
20	Benzaldehyde	100-52-7	0.350	28.31 ± 0.43a	7.79 ± 0.40b	5.15 ± 0.16c	4.69	sweet, bitter, almond, cherry
21	2-phenylethanol	60-12-8	0.140	177.3 ± 7.6a	133.6 ± 9.8b	87.86 ± 0.45c	5.70	fruity, rose, sweet, apple
22	phenylmethanol	100-51-6	0.100	116.8 ± 5.0a	59.40 ± 4.39b	12.71 ± 0.26c	5.00	floral, rose, phenol, balsamic
23	(*E*)-hex-3-en-1-ol	928-97-2	0.110	24.82 ± 0.29a	14.68 ± 0.99b	4.37 ± 0.05c	2.41	moss, fresh
24	2-[(2R,5S)-5-ethenyl-5-methyloxolan-2-yl]propan-2-ol	5989-33-3	0.320	10.74 ± 0.21a	6.52 ± 0.36b	4.94 ± 0.15c	2.06	earthy, floral, sweet, woody
25	octan-4-ol	589-62-8	0.400	12.18 ± 1.71a	2.56 ± 0.33b	0.32 ± 0.08c	3.40	-
26	1-ethyl-4-methylbenzene	622-96-8	0.040	207.7 ± 2.9a	53.16 ± 2.86b	32.77 ± 1.49c	4.37	-
27	1,3,5-trimethylbenzene	108-67-8	0.830	10.89 ± 0.15a	2.91 ± 0.15b	1.86 ± 0.08c	4.46	-
28	butylbenzene	104-51-8	0.010	8.72 ± 0.43c	119.5 ± 17.6b	223.1 ± 2.2a	2.74	-

Note: all values are mean ± SD, and different lowercase letters (a b and c) indicate significant difference at *p* < 0.05.

## Data Availability

The original contributions presented in this study are detailed within the article. For further inquiries, please direct your questions to the corresponding author.

## Supplementary Materials

The following supporting information can be downloaded at https://www.mdpi.com/article/10.3390/foods14234022/s1, Figure S1: The processing flowchart of gardenia scented black tea by traditional scenting process. Figure S2: The total ion chromatograms of GBTs and BTs obtained from GC-MS. Table S1: Instrument and equipment information. Table S2: The information of 30 volatile compounds in GBTs and BTs.
