# Supplementary material for: Aroma Characterization of Gardenia Black Tea Based on Sensory Evaluation and Headspace Solid-Phase Microextraction–Gas Chromatography–Mass Spectrometry
Source: Foods. 2025 Nov 24;14(23):4022. doi: 10.3390/foods14234022 (PMC12692459; doi:10.3390/foods14234022)
Supplement: Supplementary file 1 [file foods-14-04022-s001.zip › foods-3961413-FigS1 Table S1.pdf]

# Supplementary materials

1. **Fig. S1** The processing flowchart of gardenia scented black tea by traditional scenting process
2. **Table S1** Instrument and equipment information

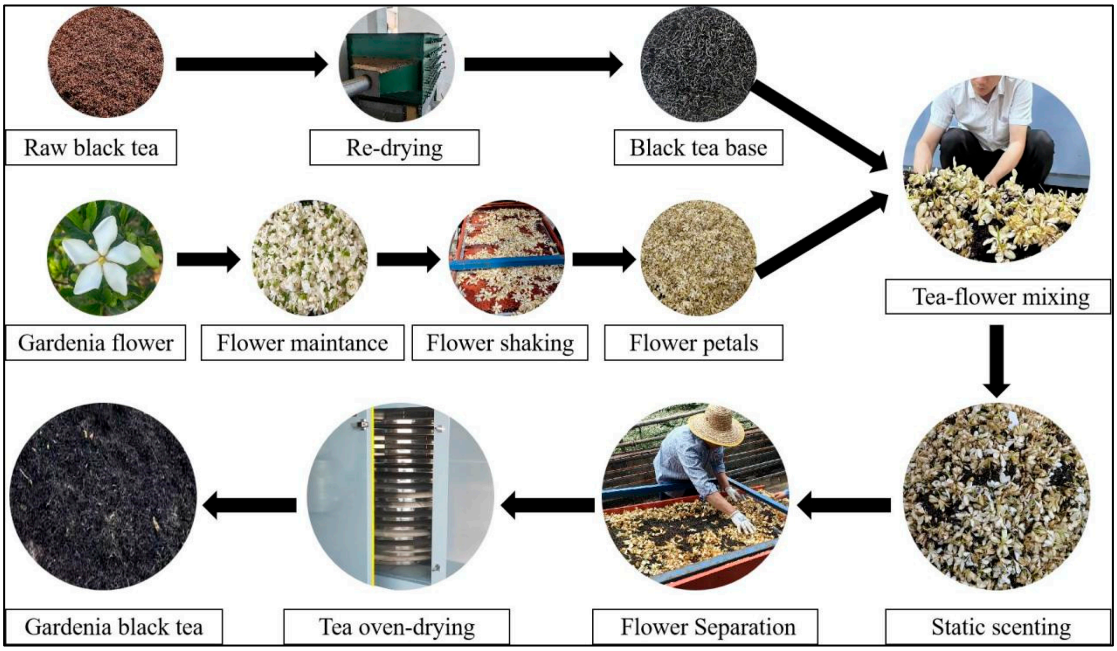


Fig. S1 The processing flowchart of gardenia scented black tea by traditional scenting process

Table1 S1 Instrument and equipment information

| Instrument and equipment | **Type and size** | **Supplier and brand** |
|---|---|---|
| GC-MS/MS | 8890-7000D | Agilent |
| Chromatographic column | DB-5MS (30 m x 0.25 mm x 0.25 μm) | Agilent |
| Ball miller | MM400 | Retsch |
| Electronic balance | MS105DU | METTLER TOLEDO |
| Extraction head | 120 μm DVB/CWR/PDMS | Agilent |
| HS-SPME | SPME Arrow | CTC Analytics AG |
| Aging device | Fiber Conditioning Station | CTC Analytics AG |
| Sample heating box | Agitator | CTC Analytics AG |
