# Supplementary material for: Aroma Characterization of Gardenia Black Tea Based on Sensory Evaluation and Headspace Solid-Phase Microextraction–Gas Chromatography–Mass Spectrometry
Source: Foods. 2025 Nov 24;14(23):4022. doi: 10.3390/foods14234022 (PMC12692459; doi:10.3390/foods14234022)
Supplement: Supplementary file 1 [file foods-14-04022-s001.zip › Table. S2 The information of 30 volatile compounds in GBTs and BT .pdf]

Table. S2 The information of 30 volatile compounds in GBTs and BTs.

Numbers	compounds	Calculated_RI	NIST_RI	CAS	Molecular weight (Da)	Formula	Class I	Odor
1	(Z)-non-6-en-1-yl acetate	1321.25	1308.00	76238-22-7	184.15	$C_{11}H_{20}O_2$	Ester	melon, honeydew, cantaloupe, green, tropical, pear, kiwi, metallic
2	1-isothiocyanato-3-methylsulfanylpropane	1321.35	1317.00	505-79-3	147.02	$C_5H_9NS_2$	Ester	earthy, vegetable, sulfury, horseradish
3	(4-methylphenyl) acetate	1164.74	1171.00	140-39-6	150.07	$C_9H_{10}O_2$	Ester	narcissus, phenol, animalic
4	[(Z)-hex-3-enyl] acetate	1005.80	1006.92	3681-71-8	142.10	$C_8H_{14}O_2$	Ester	fresh, green, sweet, fruity, banana, apple, grassy
5	methyl 2-phenylacetate	1164.85	1178.00	101-41-7	150.07	$C_9H_{10}O_2$	Ester	floral, honey, spice, waxy, sweet
6	2-phenylethyl acetate	1257.62	1260.09	103-45-7	164.08	$C_{10}H_{12}O_2$	Ester	rose, honey, tobacco
7	6-pentyloxan-2-one	1492.63	1502.45	705-86-2	170.13	$C_{10}H_{18}O_2$	Ester	creamy, coconut, fruity
8	benzyl acetate	1164.62	1167.11	140-11-4	150.07	$C_9H_{10}O_2$	Ester	sweet, floral, fruity, jasmin, fresh
9	2-phenylethyl 3-methylbutanoate	1493.00	1491.00	140-26-1	206.13	$C_{13}H_{18}O_2$	Ester	floral, fruity, sweet, rose, peach, apricot
10	propyl 2-methylbutanoate	956.57	946.00	37064-20-3	144.12	$C_8H_{16}O_2$	Ester	winey, fruity, apple, pineapple
11	3,7-dimethylocta-1,6-dien-3-ol	1097.41	1100.59	78-70-6	154.14	$C_{10}H_{18}O$	Terpenoids	floral, green

12	1,7,7-trimethylbicyclo[2.2.1]heptan-2-one	1164.43	1151.05	76-22-2	152.12	$C_{10}H_{16}O$	Terpenoids	camphor
13	5-ethyl-2-methylpyridine	1031.45	1031.00	104-90-5	121.09	$C_8H_{11}N$	Heterocyclic compound	nutty, strong, raw, potato, roasted, earthy
14	2-pentylpyrazine	1231.01	1234.00	6303-75-9	150.12	$C_9H_{14}N_2$	Heterocyclic compound	-
15	2-[(2R,5R)-5-ethenyl-5-methyloxolan-2-yl]propan-2-ol	1069.54	1075.24	34995-77-2	170.13	$C_{10}H_{18}O_2$	Heterocyclic compound	flowery
16	2-phenylethanol	1110.69	1116.10	60-12-8	122.07	$C_8H_{10}O$	Alcohol	fruity, rose, sweet, apple
17	phenylmethanol	1031.14	1034.76	100-51-6	108.06	C_7H_8O	Alcohol	floral, rose, phenol, balsamic
18	(E)-hex-3-en-1-ol	851.27	852.00	928-97-2	100.09	$C_6H_{12}O$	Alcohol	moss, fresh
19	2-[(2R,5S)-5-ethenyl-5-methyloxolan-2-yl]propan-2-ol	1069.54	1074.00	5989-33-3	170.13	$C_{10}H_{18}O_2$	Alcohol	earthy, floral, sweet, woody
20	octan-4-ol	986.50	978.00	589-62-8	130.14	$C_8H_{18}O$	Alcohol	-
21	(3E,6E)-octa-3,6-dien-2-one	1069.60	1073.00	30086-02-3	124.09	$C_8H_{12}O$	Ketone	fruity, green, grassy
22	heptan-4-one	865.19	872.00	123-19-3	114.10	$C_7H_{14}O$	Ketone	fruity, cheese, sweet, cognac, pineapple
23	1-(furan-2-yl)-2-hydroxyethanone	1085.44	1087.00	17678-19-2	126.03	$C_6H_6O_3$	Ketone	-
24	(E)-non-4-enal	1110.82	1105.00	2277-16-9	140.12	$C_9H_{16}O$	Aldehyde	fruity
25	(E)-hex-2-enal	856.85	853.20	6728-26-3	98.07	$C_6H_{10}O$	Aldehyde	green, grassy

26	2-phenylacetaldehyde	1045.14	1045.60	122-78-1	120.06	C_8H_8O	Aldehyde	floral, honey, rose, cherry
27	benzaldehyde	957.93	962.46	100-52-7	106.04	C_7H_6O	Aldehyde	sweet, bitter, almond, cherry
28	1-ethyl-4-methylbenzene	958.23	954.00	622-96-8	120.09	C_9H_{12}	Aromatics	-
29	1,3,5-trimethylbenzene	958.23	972.00	108-67-8	120.09	C_9H_{12}	Aromatics	-
30	butylbenzene	1045.37	1054.00	104-51-8	134.11	$C_{10}H_{14}$	Aromatics	-
