# Supplementary figures and images for: Aroma Characterization of Gardenia Black Tea Based on Sensory Evaluation and Headspace Solid-Phase Microextraction–Gas Chromatography–Mass Spectrometry
Source: Foods. 2025 Nov 24;14(23):4022. doi: 10.3390/foods14234022 (PMC12692459; doi:10.3390/foods14234022)

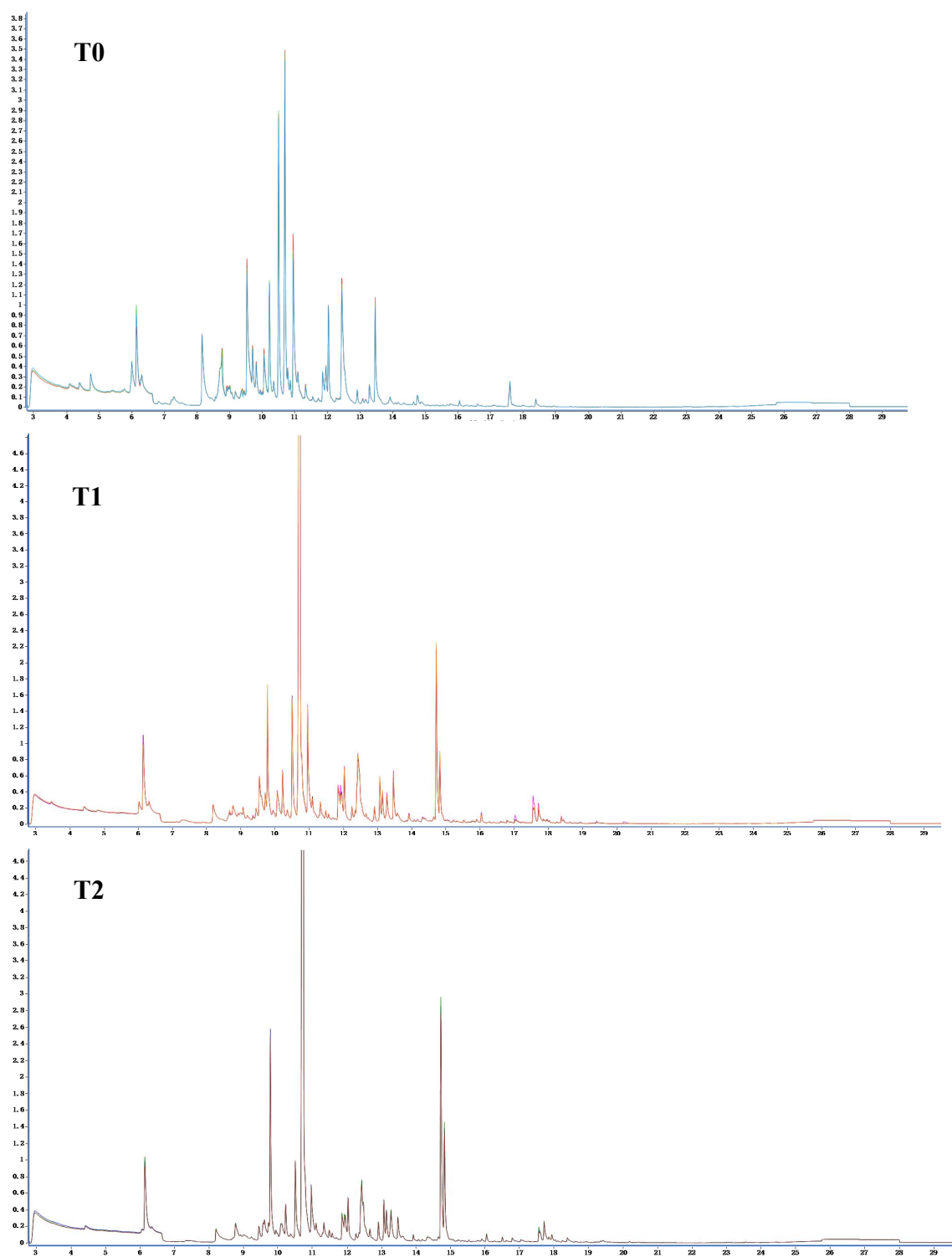

Fig. S2 The total ion chromatograms of GBTs and BTs obtained from GC-MS.

Supplement: Supplementary file 1 [file foods-14-04022-s001.zip › Figure S2. The total ion chromatograms of GBTs and BTs obtained from GC-MS..pdf]
